# Vitamin D Deficiency and Gender Alter Vasoconstrictor and Vasodilator Reactivity in Rat Carotid Artery

**DOI:** 10.3390/ijms22158029

**Published:** 2021-07-27

**Authors:** Miklós Sipos, Dóra Gerszi, Hicham Dalloul, Bálint Bányai, Réka Eszter Sziva, Réka Kollarics, Péter Magyar, Marianna Török, Nándor Ács, Mária Szekeres, György L. Nádasy, Leila Hadjadj, Eszter Mária Horváth, Szabolcs Várbíró

**Affiliations:** 1 Department of Obstetrics and Gynecology, Faculty of Medicine, Semmelweis University, Üllői Street 78/a, 1083 Budapest, Hungary; sipos.miklos.dr@gmail.com (M.S.); gerszi.dora@med.semmelweis-univ.hu (D.G.); hichamdalloul@gmail.com (H.D.); kollarics.reka@gmail.com (R.K.); torok.marianna@med.semmelweis-univ.hu (M.T.); acs.nandor@med.semmelweis-univ.hu (N.Á.); varbiro.szabolcs@med.semmelweis-univ.hu (S.V.); 2 Department of Physiology, Faculty of Medicine, Semmelweis University, Tűzoltó Street 37-47, 1094 Budapest, Hungary; banyai.balint@gmail.com (B.B.); szekeres.maria@med.semmelweis-univ.hu (M.S.); nadasy.gyorgy@med.semmelweis-univ.hu (G.L.N.); horvath.eszter@med.semmelweis-univ.hu (E.M.H.); 3 Workgroup for Science Management, Doctoral School, Semmelweis University, Üllői Street 22, 1085 Budapest, Hungary; 4 Medical Imaging Centre, Faculty of Medicine, Semmelweis University, Üllői Street 78/a, 1083 Budapest, Hungary; drmagyarpeter@gmail.com; 5 Department of Morphology and Physiology, Faculty of Health Sciences, Semmelweis University, Vas Street 17, 1088, Budapest, Hungary; 6 Department of Translational Medicine, Faculty of Medicine, Semmelweis University, Tűzoltó Street 37-47, 1094 Budapest, Hungary; leila.hadjadj@gmail.com

**Keywords:** vitamin D, vitamin D deficiency, cardiovascular disease, carotid artery, vascular reactivity, prostanoid pathway, gender, rat model

## Abstract

The vitamin-D-sensitivity of the cardiovascular system may show gender differences. The prevalence of vitamin D (VD) deficiency (VDD) is high, and it alters cardiovascular function and increases the risk of stroke. Our aim was to investigate the vascular reactivity and histological changes of isolated carotid artery of female and male rats in response to different VD supplies. A total of 48 male and female Wistar rats were divided into four groups: female VD supplemented, female VDD, male VD supplemented, male VDD. The vascular function of isolated carotid artery segments was examined by wire myography. Both vitamin D deficiency and male gender resulted in increased phenylephrine-induced contraction. Acetylcholine-induced relaxation decreased in male rats independently from VD status. Inhibition of prostanoid signaling by indomethacin reduced contraction in females, but increased relaxation ability in male rats. Functional changes were accompanied by VDD and gender-specific histological alterations. Elastic fiber density was significantly decreased by VDD in female rats, but not in males. Smooth muscle actin and endothelial nitric oxide synthase levels were significantly lowered, but the thromboxane receptor was elevated in VDD males. Decreased nitrative stress was detected in both male groups independently from VD supply. The observed interactions between vitamin D deficiency and sex may play a role in the gender difference of cardiovascular risk.

## 1. Introduction

Currently, the role of vitamin D in cardio- and cerebrovascular health is not clear, although disturbances of vitamin D homeostasis—mainly lower vitamin D levels (vitamin D insufficiency and deficiency)—play a role in atherogenesis–atherosclerosis, hypertension development and different arteriopathies (peripheral arterial diseases, aneurismal arterial diseases) [1]. The prevalence of vitamin D deficiency (25-hydroxy-vitamin D / 25(OH)D < 20 ng/mL or < 50 nmol/L [2]) is high [3] and is expected to grow worldwide; thus, adequate sun-exposure and/or medical vitamin D supplementation are increasingly important. However, a general population-based study, which investigated the intake of fat- and water-soluble vitamins, found that vitamin D intake is the least adequate in both sexes in all age groups [4].

Vitamin D deficiency is a potential risk factor for several cardio-cerebrovascular diseases and events, including stroke [5]. Impaired vitamin D signaling in functionally inactive vitamin D receptor mutant male mice caused compromised cerebrovascular adaptation to unilateral carotid artery occlusion [6]. Four-week-long vitamin D deficient and vitamin D toxic (25(OH)D > 200 ng/mL or > 500 nmol/L [2]) diets resulted in significantly decreased carotid artery diameter and significantly enhanced wall thickness in male rats [7]. Patients with ischemic stroke had significantly lower 25(OH)D levels than control ones and, according to multiple logistic regression, vitamin D considered as a significant predictor in stroke patients and vitamin D deficiency is associated with ischemic stroke [8]. The National Health and Nutrition Examination Survey data evaluation also showed that vitamin D deficiency may be associated with increased stroke risk while higher 25(OH)D levels are associated with reduced stroke risk and these associations were pronounced in the age group of 20–50-year-old women. Moreover, people who have previously had stroke had significantly lower 25(OH)D levels than controls [9]. Among elderly people, a significant linkage was shown between serum 25(OH)D levels and carotid artery distensibility and intima-media thickness (IMT), with these results referring to the possible effects of vitamin D on the functional and structural properties of carotid artery [10]. Suboptimal vitamin D levels (25(OH)D < 30 ng/mL or < 75 nmol/L [2] ) and vitamin D deficiency are associated with carotid plaque thickness and with the presence and volume of carotid intraplaque hemorrhage, a parameter which may be a better predictor of unstable plaque and a better estimate of recurrent stroke risk [11].

Stroke is one of the main life-threatening cardio-cerebrovascular and neurological illnesses and its risk also differs between males and females. A retrospective cross-sectional study among stroke patients found that the incidence of stroke and prevalence of stroke risk factors (hypertension, heart diseases, diabetes and hyperlipidaemia) are higher in males than in females [12]. 

The cerebrovascular consequences of vitamin D deficiency may also be influenced by gender. In healthy men, but not in women, higher total vitamin D intake was associated with decreased cardiovascular disease (CVD) risk [13]. According to the MONICA study, middle-aged women with low (<51.45 nmol/L) 25(OH)D levels had increased risk for stroke and higher total CVD and all-cause mortality during a 17-year-follow-up [14]. Type 2 diabetic patients with carotid atherosclerotic plaque had significantly lower 25(OH)D levels than the control group and 25(OH)D concentrations were inversely correlated with carotid intima-media thickness (CIMT) in men, but not in women [15]. 

Previous results from our research group also suggest that gender-specific alterations of vascular function and structure can be observed in the renal and cerebral arteries of vitamin D deficient rats [16,17]. In the anterior cerebral artery, vitamin D deficiency resulted in increased wall thickness and testosterone-induced contraction only in male rats [16]. In renal arteries vitamin D deficiency led to impaired acetylcholine (Ach)-induced relaxation in both genders, whereas increased phenylephrine contraction was only found in male animals. Vascular function measurements in the presence of cyclooxygenase (COX) inhibitor and the immunohistochemical labeling of endothelial nitric oxide synthase (eNOS) in these vessels suggest that eNOS and prostanoid pathways may play a role in the gender-specific vascular dysfunction in vitamin D deficiency [17].

Carotid arterial function plays an important role in the regulation of cerebral blood flow and systemic blood pressure by influencing the sensitivity of the high pressure baroreceptor reflex. Monitoring the in vivo condition and characteristics of carotid artery, such as CIMT and carotid plaque area (PA) measurements, are important tools for cardio- and cerebrovascular risk assessment [18]. 

In the present study, our aim was to examine the possible gender-specific effect of vitamin D deficiency on carotid arteries of rats. The potential role of elastic and contractile elements, eNOS and COX enzymes and a nitrative stress marker were also investigated. 

## 2. Results

### 2.1. Vascular Function of Carotid Arteries

#### 2.1.1. Phenylephrine-Induced Contraction of Carotid Arteries

**Figure 1:**
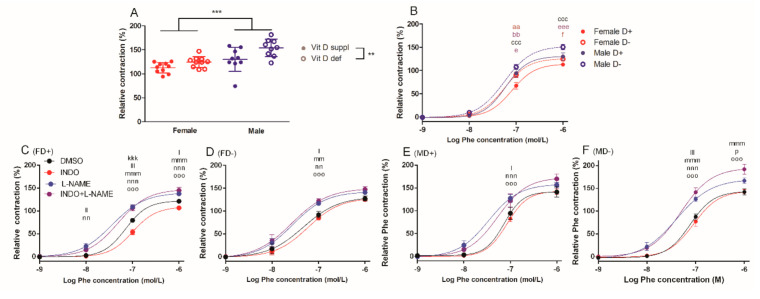
Contraction ability of isolated carotid artery segments: (**A**) phenylephrine (Phe)-induced contraction in the four experimental groups at 10^−6^ mol/L Phe concentration. Male gender and vitamin D deficiency were associated with more pronounced relative contraction. Data are shown as individual data points; horizontal lines represent mean ± SD. Two-way ANOVA; factors: gender, vitamin D status. **: *p* < 0.01, ***: *p* < 0.001. (**B**) Phe-induced contraction. Male rats showed significantly increased contraction compared to females at Phe concentration of 10^−7^ mol/L independently from vitamin D status. Vitamin D deficient female vessels had stronger contraction compared to their vitamin D supplemented counterparts. At Phe concentration of 10^−6^ mol/L, MD− arteries contracted more than FD− ones. Vitamin D deficient male rats showed increased contraction compared to their vitamin D supplemented counterparts. Data are shown as mean ± SEM; *n* = 9–11 in each group; repeated measures ANOVA, Bonferroni’s post hoc test; aa: *p* < 0.01 FD+ vs. FD−, bb: *p* < 0.01 FD+ vs. MD+, ccc: *p* < 0.001 FD+ vs. MD−, e: *p* < 0.05 FD− vs. MD−, eee: *p* < 0.001 FD− vs. MD−, f: *p* < 0.05 MD+ vs. MD−. Phe-induced contractions in the presence of L-NAME and/or indomethacin (INDO) or their vehicle DMSO (**C**) in female vitamin D supplemented rats (FD+) (**D**) in female vitamin D deficient rats (FD−), (**E**) in male vitamin D supplemented rats (MD+) and (**F**) in male vitamin D deficient rats (MD−). L-NAME increased the level of contraction in all experimental groups. Co-incubation with INDO further augmented the contraction only in MD− rats. INDO itself decreased the degree of vascular reaction only in the FD+ group. Data are shown as mean ± SEM; *n* = 5–11 in each group; repeated measures ANOVA, Bonferroni’s post hoc test; kkk: *p* < 0.001 DMSO vs. INDO, l: *p* < 0.05 DMSO vs. L-NAME, ll: *p* < 0.01 DMSO vs. L-NAME, lll: *p* < 0.001 DMSO vs. L-NAME, mm: *p* < 0.01 DMSO vs. INDO+L-NAME, mmm: *p* < 0.001 DMSO vs. INDO+L-NAME, nn: *p* < 0.01 INDO vs. L-NAME, nnn: *p* < 0.001 INDO vs. L-NAME, ooo: *p* < 0.001 INDO vs. INDO+L-NAME, p: *p* < 0.05 L-NAME vs. INDO+L-NAME.

Gender-specific difference was observed in the phenylephrine-induced contraction at 10^−6^ mol/L phenylephrine concentration and male gender was associated with more pronounced contraction independently from vitamin D status. Generally, vitamin D deficiency resulted in increased level of phenylephrine-induced contraction (Figure 1A). At 10^−7^ mol/L phenylephrine concentration, male vitamin D deficient vessels showed increased reaction, compared to their female counterparts. Furthermore, vitamin D deficient female arteries were more reactive than female vitamin D supplemented ones. At 10^−6^ mol/L phenylephrine concentration, vessels of vitamin D deficient male rats showed stronger contraction than those of the vitamin D supplemented male animals (Figure 1B).

In order to explore the role of endothelial nitric oxide synthase (eNOS) and cyclooxygenases in the observed reactivity differences, phenylephrine-induced contraction was repeated in the presence of N(G)-Nitro-L-arginine methyl ester/L-NAME and indomethacin and their combination. L-NAME increased the degree of contraction in all experimental groups. However, indomethacin failed to alter the observed reactivity in the presence of L-NAME in most groups, except the vitamin D deficient males, where indomethacin further increased the contraction compared to L-NAME alone. Indomethacin itself decreased the level of contraction only in the female vitamin D supplemented group, suggesting the significant involvement of constrictor prostanoids in these animals (Figure 1C–F). The lack of indomethacin effect in the vitamin D supplemented male group and in the vitamin D deficient female group may indicate gender and vitamin D dependent regulation of prostanoid effects in vascular contractility.

#### 2.1.2. Acetylcholine-Induced Relaxation of Carotid Arteries

**Figure 2:**
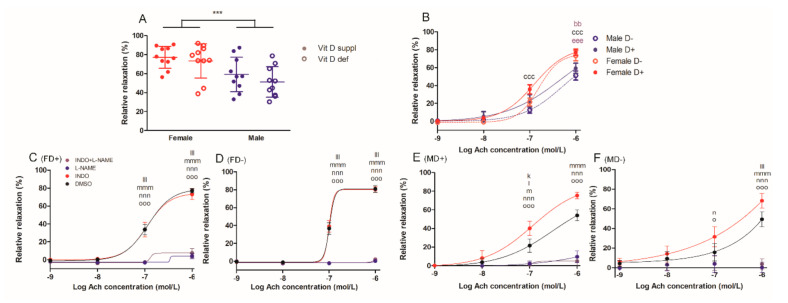
Relaxation ability of isolated carotid artery segments: (**A**) acetylcholine (Ach)-induced relaxation in the four experimental groups at 10^−6^ mol/L Ach concentration. Male gender was associated with less pronounced relative relaxation. Data are shown as individual data points; horizontal lines represent mean ± SD. Two-way ANOVA; factors: gender, vitamin D status. ***: *p* < 0.001. (**B**) Ach-induced relaxation. Male rats showed significantly reduced relaxation compared to females at Ach concentration of 10^−6^ mol/L independently from vitamin D status. Data are shown as mean ± SEM; *n* = 9–11 in each group; repeated measures ANOVA, Bonferroni’s post hoc test; bb: *p* < 0.01 FD+ vs. MD+, ccc: *p* < 0.001 FD+ vs. MD−, eee: *p* < 0.001 FD− vs. MD−. Ach-induced relaxation in the presence of L-NAME and/or indomethacin (INDO) or their vehicle DMSO (**C**) in female vitamin D supplemented rats (FD+), (**D**) in female vitamin D deficient rats (FD−), (**E**) in male vitamin D supplemented rats (MD+) and (**F**) in male vitamin D deficient rats (MD−). L-NAME blocked the vasodilation in all experimental groups, co-incubation with INDO had no additional effect. INDO itself increased the degree of relaxation only in both male groups. Data are shown as mean ± SEM; *n* = 6–11 in each group; repeated measures ANOVA, Bonferroni’s post hoc test; k: *p* < 0.05 DMSO vs. INDO, l: *p* < 0.05 DMSO vs. L-NAME, m: *p* < 0.05 DMSO vs. INDO+L-NAME, n: *p* < 0.05 INDO vs. L-NAME, lll: *p* < 0.001 DMSO vs. L-NAME, mmm: *p* < 0.001 DMSO vs. INDO+L-NAME, nnn: *p* < 0.001 INDO vs. L-NAME, o: *p* < 0.05 INDO vs. INDO+L-NAME, ooo: *p* < 0.001 INDO vs. INDO+L-NAME.

Gender difference was also observed in the acetylcholine-induced relaxation of carotid artery segments at 10^−6^ M acetylcholine concentration (Figure 2A). Vitamin D deficiency failed to alter this function of the vessels in both genders. At this acetylcholine concentration, vitamin D supplemented female vessels had more pronounced relaxation compared to both male groups, while vitamin D deficient females only differed from vitamin D deficient male counterparts (Figure 2B). Nitric oxide inhibition with L-NAME successfully blocked the acetylcholine-induced vasodilation in all experimental groups, while co-incubation with indomethacin had no additional effect. In both male groups, indomethacin itself significantly increased the endothelium-induced relaxation (Figure 2C–F).

### 2.2. Histological Changes of the Carotid Arteries

**Figure 3:**
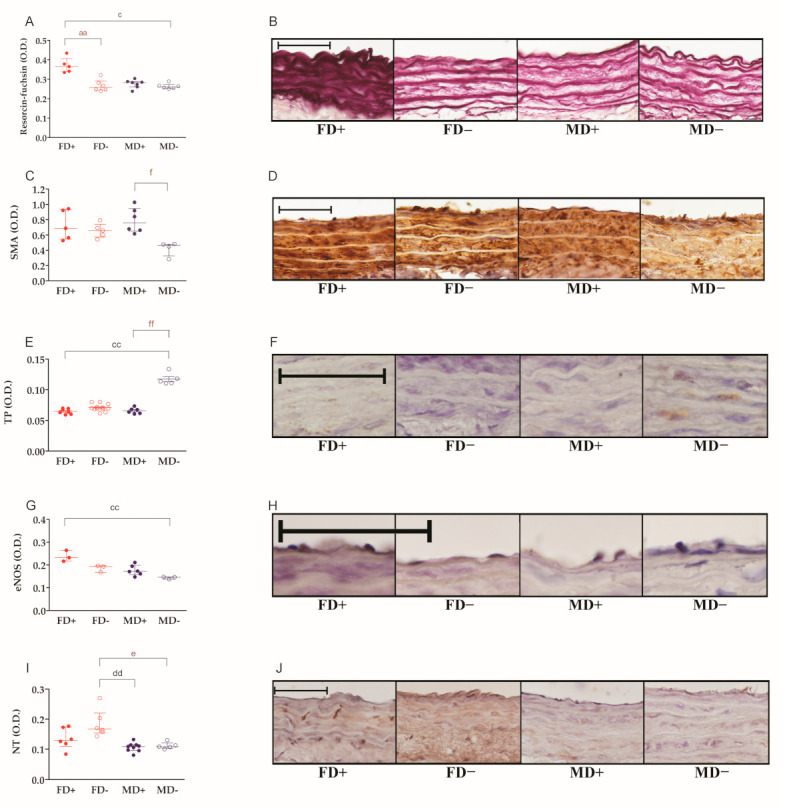
Histological changes of the carotid arteries: (**A**) elastic fiber density of carotid artery segments. Tissue sections were stained by the purple-colored resorcin-fuchsin stain. Vitamin D deficient female and male arteries showed significantly lower optical density than vitamin D supplemented female vessels. (**B**) Representative images of resorcin-fuchsin-stained carotid artery sections. (**C**) Alpha smooth muscle actin (SMA) immunohistochemical labeling intensity in the media layer of carotid arteries. In vitamin D deficient male rats, the measured optical density was significantly lower compared to vitamin D supplemented male animals. (**D**) Representative images of carotid arteries stained against SMA. (**E**) Thromboxane A2 receptor (TP) density of carotid arteries. Vitamin D deficient male rats showed higher receptor density compared to vitamin-supplemented male and female animals. (**F**) Representative images of TP-stained vessels. (**G**) Optical density of eNOS labeling in the intimal layer of carotid arteries. Vitamin D deficiency induced a significant decrease in eNOS staining intensity in males. (**H**) Representative images of vessels labeled with anti-eNOS antibody. (**I**) 3-Nitrotyrosine (NT) staining intensity of carotid arteries. Both male groups showed lower positivity than vitamin D supplemented female rats. (**J**) Representative images of NT-stained carotid artery sections. Specific immunohistochemical labeling was visualized by the brown-colored diamino-benzidine (DAB), while blue-colored hematoxylin served as counterstaining. Scale bars show 50 µm. Data are presented as individual data points and lines represent the median [IQR]; Kruskal–Wallis test with Dunn’s post hoc test; aa: *p* < 0.01 FD+ vs. FD−, c: *p* < 0.05 FD+ vs. MD−, cc: *p* < 0.01 FD+ vs. MD−, dd: *p* < 0.01 FD− vs. MD+, e: *p* < 0.05 FD− vs. MD−, f: *p* < 0.05 MD+ vs. MD−, ff: *p* < 0.01 MD+ vs. MD−.

The density of elastic fibers in the isolated carotid arteries was significantly decreased by vitamin D deficiency in female rats, but not in males. The marked, but not significantly lower, elastic fiber density of male carotid arteries may have played a role in this phenomenon. On the other hand, the staining intensity observed after the immunolabeling of SMA was decreased by vitamin D deficiency in male, but not in female, carotid arteries. Vitamin D deficiency induced an increase in the immune positivity of thromboxane A2 receptor only in male animals. The optical density of eNOS immunostaining was altered by neither gender nor vitamin D deficiency. Nitrative stress, represented by tyrosine nitration, was slightly lower in both male groups compared to vitamin D deficient female samples (Figure 3A–J). 

## 3. Discussion

The main findings of the present study were the following: (i) the carotid artery, which has a primary role in cerebral blood flow and systemic blood pressure regulation, shows gender differences in its reaction to both vasoconstrictor and vasodilator agents; (ii) vitamin D deficiency causes vascular injury in both sexes; and (iii) gender differences can be observed in the pathomechanism of vascular injury caused by vitamin D deficiency.

Recent studies suggest that the vascular function of the carotid artery is influenced by gender. Significantly higher serotonin-induced vasoconstriction was found in male mice compared to females [19]. In addition, these sex-specific alterations are also influenced by the type of vessel under investigation. The release of the cyclooxygenase (COX)-derived constricting factors in mesenteric arterial rings is more pronounced in male spontaneous hypertensive rats (SHR), than their female counterparts [20]. In male rats the sensitivity to endothelin-1‘s vasoconstrictor effect on coronary resistance arteries is significantly higher than in females [21]. In males the myogenic tone and the reaction to thromboxane A2 agonist are also more pronounced on coronary resistance arteries [22]. On the contrary, no gender differences were found in renal arteries for Phe-induced contraction [17], and there were also no gender differences in endothelium-dependent vasoconstriction of the popliteal artery [23]. In our current study, we found significantly higher phenylephrine-induced vasoconstriction on carotid arterial rings of male animals independently from their vitamin D status. 

Endothelium-dependent vasodilation is also influenced by gender, corresponding to the higher cardiovascular risk of men. The effect of sex hormones on the expression of enzymes involved in the synthesis of endothelium-derived relaxation factors, like eNOS, COX-1 and 2 and prostacyclin synthase, is believed to play a role in this phenomenon [24]. Acetylcholine-induced relaxation on carotid artery rings was found to be significantly higher in SHR female animals than in males, while, in parallel, indomethacin caused increased relaxation in males, while failing to induce change in young females [25]. In our present study, we found no gender difference in eNOS expression of carotid arteries. Our observation that, while in male rats general COX inhibition increased the acetylcholine-induced relaxation, it failed to influence this function in females, suggests that gender-specific alterations of vasoactive prostanoids’ production or action may have played a role in the reduced endothelium-dependent relaxation of the investigated vessels. On the other hand, in the present study, we found no gender difference in TP-specific staining intensity of carotid arteries in vitamin D supplemented rats. 

Overall, the weakest vasodilation occurred in the vitamin D deficient male group that was accompanied by the reduced immunohistochemical labeling of eNOS. The production of oxygen-derived free radicals, especially superoxide, may reduce the bioavailability of nitric oxide through their spontaneous reaction forming the potent oxidant peroxynitrite [26]. A characteristic reaction of nitrogen-derived free radicals, especially peroxynitrite, is the nitration of protein tyrosine residues (NT). On the other hand, we could not show the role of this phenomenon in the reduced endothelium-dependent relaxation induced by male gender. NT positivity was significantly lower in vitamin D deficient male coronary arteries compared to their female counterparts. The known antioxidant effect of testosterone may have contributed to this observation [27].

The risk and the severity of stroke correlate to vitamin D deficiency [28,29]. The increased arterial stiffness that can be observed in vitamin D deficiency may contribute to the increased cardiovascular risk, which can be markedly reduced by vitamin D supplementation (>2000 IU/day) in both sexes [30]. Vitamin D supplementation can dose-dependently decrease arterial stiffness in overweight, vitamin D deficient male and female African Americans [31]. Vitamin D deficiency also contributes to the atherosclerotic transformation of the carotid artery [32]. In our recent study we found that vitamin D deficiency increased the degree of phenylephrine-induced contraction in both sexes. The weakest vasoconstriction was found in female vitamin D supplemented animals. In vitamin D deficient females, increased vasoconstriction was observed that demolished the observed gender difference among the vitamin D supplemented animals. The strongest vasoconstriction was seen in vitamin D deficient males. Similar observations were described in other vessel types. As the consequence of vitamin D deficiency, the myogenic tone of the mesenteric arteries doubles [33]. In coronary arteries of male animals, vitamin D deficiency decreased the vascular reactivity to thromboxane A2 and sexual steroids (estrogen and testosterone) [34]. On the other hand, in renal arteries, we saw increased Phe-induced contraction only in female, but not in male rats [17,26]. Vitamin D can inhibit TP receptor expression [35] and the increased TP immunolabeling intensity observed in vitamin D deficient males may reflect the lack of this suppressing effect, contributing to their increased Phe-induced contraction. Estrogen was also shown to attenuate TP expression [36] that may play a role in the preserved TP expression of vitamin D deficient female carotid arteries. These observations suggest that the consequences of vitamin D deficiency are influenced by gender and the type of the investigated vessel.

Our recent data show that in the carotid arteries of female animals, the balance of the produced prostanoids shifts to vasoconstrictors, as general COX inhibition leads to decreased Phe-induced contraction, which is demolished by vitamin D deficiency. Male vessels also had vasoconstrictor dominance, as indomethacin caused increased Ach-induced relaxation; however, it was not altered by vitamin D deficiency. Interestingly, when indomethacin was applied together with L-NAME, it augmented the level of Phe-induced contraction in vitamin D deficient males. The already described cross-talk between eNOS and COX systems, which was not observable in vitamin D supplemented rats, may have been augmented in this experimental group. In rats fed with standard diet, no interaction was found between these two signaling pathways in the regulation of cerebrocortical microcirculation [37].

As for vasorelaxation, similarly to vasoconstriction, gender differences were observed that might have been affected by the type of the examined vessel. Flow-mediated dilation (FMD) of the brachial artery is not gender-related, while the FMD of the popliteal artery is significantly higher in physically active adult women [38]. Nitroglycerine-mediated dilation is also lower in men’s brachial artery [39]. In our current study, we found gender differences in acetylcholine-induced relaxation on carotid arterial rings: male animals showed less relaxation compared to females regardless of their vitamin D status.

Differences in vasoconstriction and vasodilation responses of coronary arteries in this study may be also explained by the observation that both smooth muscle elements and elastic fibers are altered by gender and vitamin D deficiency. The amount of elastic fibers of resistance coronary arteries is significantly lower in male rats than in females [40]. Previous studies examining the effect of vitamin D deficiency on elastic elements showed that abdominal aortic aneurysms have significantly lower levels of elastin in the intima-media composites of male aneurysm walls than of females [41]. Moreover, serious vitamin D deficiency impairs the elastic quality of aorta [42], while in the cerebral arteries this kind of change cannot be observed, not even in vitamin D deficiency [43]. So, the effect of vitamin D deficiency on elastic fibers can also depend on the type of the investigated vessel. In the present study, we found decreased elastic fiber density in vitamin D deficient male and female carotid arteries, whereas alpha smooth muscle actin immunolabeling was the weakest in the vitamin D deficient male rats. Vitamin D has been previously shown to increase SMA expression in the aortae of rats on a high fat diet [44], which may have played a role in the changes observed in SMA density.

Limitations and strengths: According to our current knowledge, this is the first study which investigated the ex vivo vascular function, histological characteristics and gender differences of female and male rat carotid artery in response to a vitamin D deficient state. The small sample size hindered out investigation of other possibly affected vascular reactivity pathways. The lack of a naive animal group with only a normal diet was another limitation of our study. Further thorough, basic translational and clinical research is needed to clarify the connection between vitamin D deficiency, functional and histological cerebrovascular pathology and its gender difference.

## 4. Materials and Methods

### 4.1. Chemicals

Ex vivo vascular functional measurements on isolated rat carotid arteries were performed in Krebs–Ringer solution (in mmol/L: NaCl 119, KCl 4.7, NaH_2_PO_4_ 1.2, MgSO_4_ 1.17, NaHCO_3_ 24, CaCl_2_ 2.5, glucose 5.5 and EDTA 0.034). The solution was freshly prepared, stored at 37 °C, and bubbled (gas mixture composed of O_2_ 20%, CO_2_ 5% and N_2_ 75%) to maintain stable pH. Chemicals were purchased from Sigma-Aldrich (Sigma-Aldrich, St. Louis, MO, USA–Budapest, Hungary).

### 4.2. Animals

The study was performed in accordance with the Guide for the Care and Use of Laboratory Animals published by the US National Institutes of Health (8th edition, 2011) and the EU-conforming Hungarian Law on Animal Care (XXVIII/1998). The institutional Animal Care Commission has confirmed the research protocol (IRB: 8/2014 PEI/001/1548-3/2014, PEI/001/820-2/2015).

A total of 48 adolescent (21–28 day old) male and female Wistar rats, weighing 100–140 g, were delivered to the Animal Facility of Semmelweis University in agreement with Charles River (Charles River Ltd., AnimaLab, Vác, Hungary). Rats of both genders were randomly assigned to two further groups, and as a result, four experimental groups were obtained: a female vitamin D supplemented group (FD+), a female vitamin D deficient group (FD−), a male vitamin D supplemented group (MD+) and a male vitamin D deficient group (MD−); *n* = 11–13 in each group.

### 4.3. Chronic Treatment of the Rats

In order to induce vitamin D deficiency via reduced intake, rats in the corresponding groups were fed ad libitum with vitamin D Free Lab rat/mouse chow (Ssniff Spezialdiaten GmbH, Soest, Germany) containing less than 5 IU/kg vitamin D for eight weeks resulting in vitamin D deficiency (below 10 ng/mL) [43,45]. Rats in vitamin D supplemented groups were fed ad libitum with a normal chow containing 1000 IU/kg of vitamin D. Furthermore, oral administration of additional vitamin D through a gavage cannula was applied to ensure the targeted plasma vitamin D levels [43,45]: 500 IU cholecalciferol on the second week and a weekly maintenance dose of 140 IU/100g on the fourth, fifth, sixth and seventh weeks (Vigantol (cholecalciferol) 20,000 IU/mL, Merck/Merck Serono, Darmstadt, Germany). The treatment protocol resulted in four experimental groups; vitamin D supplemented female and male (FD+, MD+) and vitamin D deficient female and male (FD−, MD−) groups. The animals had access to tap water ad libitum. Rats were housed at constant room temperature (22 ± 1 °C) with a 12 h/12 h light–dark cycle. All experimental groups had normal blood pressure [45,46]. Serum 25(OH)D levels of the animals were the following: FD+: 32.328 ± 4.49 ng/mL; FD−: 6.044 ± 0.63 ng/mL; MD+: 19.66 ± 0.81 ng/mL; MD− rats: 3.59 ± 0.21 ng/mL [43,45]. Final body weight, weight gain and serum testosterone levels were not significantly influenced by vitamin D status in either gender [43,45,46].

After 8 weeks, rats were anesthetized with Nembutal (45 mg/kg intraperitoneal (i.p.)), perfused with heparinized nKR solution for 2 min. Carotid arterial segments were cut into five equal rings (2 mm long), four of which were placed on a conventional wire myograph setup, while the fifth vascular ring was fixed in formaldehyde and embedded in paraffin.

### 4.4. Myography

A conventional wire myograph system was adopted to measure the isometric tension of isolated carotid arterial rings (610-M MultiMyograph System, Danish Myo Technology, Aarhus, Denmark, with Lab-Chart Evaluation System, AD Instruments, Oxford, UK–Ballagi Ltd., Budapest, Hungary). The organ chambers were filled with nKR solution, kept at 37 °C and bubbled (gas mixture composed of O_2_ 20%, CO_2_ 5% and N_2_ 75%) to maintain stable pH. Following the development of stable pre-tension (15 mN), the contractility of the vessels was obtained when applying 124 mmol/L K^+^, which served as the reference value of the contraction forces. Vascular rings were equilibrated in nKR, and accumulative doses of phenylephrine (10^−9^–10^−6^ mol/L) were administrated to induce contraction. Acetylcholine-induced vasodilation was examined by incubating the vessels with increasing doses of Ach (10^−9^–10^−6^ mol/L), subsequent to Phe precontraction (10^−6^ mol/L). Measurement of Phe-induced contraction and Ach-induced vasodilation was repeated in the presence of the nitric oxide synthase inhibitor N(G)-Nitro-L-arginine methyl ester (L-NAME) (10^−4^ mol/L) and the general COX inhibitor indomethacin (10^−5^ mol/L) or their vehicle DMSO.

### 4.5. Immunohistochemistry

Carotid arterial tissue sections were stained with resorcin-fuchsin (RF). Immunohistochemistry was performed to label alpha smooth muscle actin (SMA), thromboxane A2 receptor (TP), endothelial nitric oxide synthase (eNOS) and 3-nitrotyrosin (NT). Antigen retrieval was performed by heating the slides in citrate buffer (pH = 6) following deparaffinization. Then, 3% H_2_O_2_ in dH_2_O was applied to block endogenous peroxidase activity. Non-specific labeling was prevented via utilization of 2.5% normal horse serum (Vector Laboratories, Burlingame, CA, USA). Primary antibodies (SMA: 1:10,000; eNOS: 1:50 (Abcam, ab46545 and ab76198, Cambridge, UK)), TP 1:50 (MyBioSource, MBS2032166, San Diego, CA, USA) and NT 1:500 (Merck Millipore–Sigma-Aldrich, 06-284, Budapest, Hungary) were applied overnight at 4 °C. For SMA and eNOS horseradish-peroxidase-linked anti-mouse was used for secondary labeling, while for TP and NT anti-rabbit polyclonal horse antibody (Vector Laboratories, MP-7402 or MP-7401, Burlingame, CA, USA) was used for secondary labeling. Visualization of specific labeling was accomplished by brown-colored diamino-benzidine (DAB) (Vector Laboratories, SK-4100, Burlingame, CA, USA), while blue-colored hematoxylin served for counterstaining (Vector Laboratories, H-3404-100, Burlingame, CA, USA). Brightfield microscopy images were acquired using a Nikon ECLIPSE NI-U microscope and Nikon DS-Ri2 camera (Nikon Corporation, Minato City, Tokyo, Japan) with 40x objective. Non-calibrated optical density of the media layer of resorcin-fuchsin-stained vessels was obtained for the purpose of assessing the density of non-contractile elements using ImageJ software (National Institutes of Health (NIH), Bethesda, MA, USA). The measurements of the non-calibrated optical density of specific staining in the intimal or medial layers of the vessel wall were also completed with ImageJ software in the case of immunohistochemical labeling.

### 4.6. Statistical Analysis

Repeated measures two-way ANOVA was completed using Bonferroni’s post hoc test with GraphPad Prism 8 (GraphPad Software, San Diego, CA, USA) for the analysis of vascular function curves. At a certain agonist concentration, vascular reactivity was analyzed by two-way ANOVA (gender, vitamin D status). The Kruskal–Wallis test with Dunn’s multiple comparison test was applied to accomplish the comparison of histological and immunohistochemical evaluations of the experimental groups. *p* < 0.05 was uniformly accepted as the threshold for statistical significance. Data are presented as the mean ± SEM or median [IQR]. 

Significance symbols (Table 1):

## 5. Conclusions

Differences in cerebral blood flow, as well as gender and vitamin D dependent differences of stroke risk, can be partially explained by the local changes of circulatory control. In the present study, vitamin D deficiency resulted in vascular impairments in both genders, although gender differences in the pathomechanism could be observed. Both vitamin D deficiency and male gender resulted in increased contraction of carotid artery. Decreased relaxation reactivity of the carotid arteries was observed in male gender independently from their vitamin D status. Inhibition of prostanoid signaling reduced contraction in females, but increased relaxation ability in male rats. These functional changes were accompanied by vitamin D and gender-specific structural and protein expression alterations, which were also characteristic of the examined vessel type, the carotid artery.

## Figures and Tables

**Table 1:** The number of the symbols (x) indicates the strength of the significance: x: *p* < 0.05, xx: *p* < 0.01; xxx: *p* < 0.001. The meanings of the letter colors are as follows: gender difference is indicated by violet (**b**,**e**), while burgundy highlights a significant difference through different vitamin D statuses (**a**,**f**).

Between the Groups		Between the Inhibitors	
a:	FD+ vs. FD−	k:	DMSO vs. INDO
b:	FD+ vs. MD+	l:	DMSO vs. L-NAME
c:	FD+ vs. MD−	m:	DMSO vs. INDO+L-NAME
d:	FD− vs. MD+	n:	INDO vs. L-NAME
e:	FD− vs. MD−	o:	INDO vs. INDO+L-NAME
f:	MD+ vs. MD−	p:	L-NAME vs. INDO+L-NAME
